# Sailing towards health: HIV/AIDS awareness, comprehensive knowledge, prevention methods, and information sources among fishers in Elmina, Ghana

**DOI:** 10.1186/s12889-026-29040-2

**Published:** 2026-08-17

**Authors:** Margaret Appiah, Sylvester Kyei-Gyamfi, Frank Kyei-Arthur, Theophilus Kwabena Abutima, Myles Ongoh

**Affiliations:** 1 Department of Environment and Public Health, University of Environment and Sustainable Development, Somanya, Ghana; 2 Department of Children, Ministry of Gender, Children and Social Protection, Accra, Ghana; 3 Department of Sociology and Anthropology, University for Development Studies, Nyankpala Campus, Nyankpala, Ghana; 4 LEAP Management Secretariat, Ministry of Gender, Children and Social Protection, Accra, Ghana

**Keywords:** Awareness, Comprehensive knowledge, Elmina, Fishers, Ghana, HIV/AIDS, Prevention methods

## Abstract

**Background:**

Fishers are more vulnerable to HIV infections due to their high mobility, transactional sex, and socio-economic vulnerabilities. However, fishers often have poor knowledge of HIV. This study examined HIV/AIDS awareness and comprehensive knowledge among fishers in Elmina, Ghana. It also investigated fishers’ knowledge of HIV prevention methods and information sources.

**Methods:**

This study used a convergent parallel mixed-methods design, involving 385 respondents for the quantitative data and 30 key informants and 2 focus group discussions for the qualitative data. Descriptive and inferential statistics were used to analyze quantitative data, and thematic analysis guided the qualitative data analysis.

**Results:**

The study found that although 97.9% of respondents had heard of HIV/AIDS, 44.4% demonstrated low comprehensive knowledge of the disease. There were gaps in fishers’ knowledge about HIV/AIDS, especially HIV prevention methods and myths about HIV transmission. Radio and television were the main sources of HIV/AIDS knowledge among fishers.

**Conclusions:**

The findings of this study highlight the need for policymakers to tailor HIV/AIDS sensitization efforts to bridge fishers’ HIV/AIDS knowledge gap to reduce their risks of HIV/AIDS infection. Furthermore, policymakers can leverage radio and television to convey their HIV/AIDS messages effectively.

**Supplementary Information:**

The online version contains supplementary material available at https://doi.org/10.1186/s12889-026-29040-2.

## Background

Since the outbreak of the human immunodeficiency virus (HIV), it has infected 84.2 million people and killed roughly 40.1 million globally. By the end of 2021, 38.4 million people worldwide were HIV positive. Even though the impact of the epidemic continues to differ greatly between different countries and areas, an estimated 0.7% of adults in the world between the ages of 15 and 49 are HIV positive. Africa continues to be the region with the highest burden, with about one in every twenty-five adults (3.4%) infected with HIV, accounting for more than two-thirds of all HIV-positive individuals worldwide [45].

Fishers are one of the population groups with an HIV/AIDS prevalence greater than the general population, and they have been identified as being at the highest risk [10, 26, 35, 38]. The fishing industry is often characterized by high mobility, transactional sex, and socio-economic vulnerabilities that increase the risk of HIV infection. These conditions make fishers particularly susceptible to risky sexual behaviors, increasing their vulnerability to the virus [19, 35]. Evidence indicates that fishers often have poor knowledge of HIV, and the fisheries literature is replete with examples of how poorly informed fishers and fishing communities are about HIV [10, 50]. Earlier research attributes this lack of knowledge of HIV to the significant mobility of fishers [50]. High mobility limits fishers’ access to sustained HIV education programs, making it difficult for them to receive consistent and accurate information about HIV prevention, treatment, and transmission [29]. Additionally, most fishers have poor educational backgrounds, with the bulk of them either being illiterate or school dropouts, which further explains their inadequate knowledge of HIV [28].

Consequently, efforts have been made to strengthen the knowledge of HIV among fishers worldwide, leading to significant declines in infections in some countries. For example, Thailand and Uganda have employed several strategies focused on behavioral change, specifically the promotion of faithfulness, abstinence, consistent condom use, and sexual partner reduction, which contributed to the decline in HIV incidence in the two countries [25]. Some fishing communities in Africa have equally experienced significant declines following the promotion of sexual behavior change [25]. Despite these efforts, disparities exist in how different fishing communities access and use HIV-related information. The effectiveness of various interventions depends on socio-cultural factors, literacy levels, and the reliability of information sources available to fishers.

Even though there have been numerous HIV programs for fishers, earlier studies of fishing communities in Elmina revealed that some of them have inaccurate knowledge and misconceptions about the virus and disease [22, 28]. Erroneous beliefs about HIV transmission persist among some fishers, who believe that HIV can spread through supernatural means, mosquito bites, or food sharing. Such myths hamper effective prevention efforts and contribute to the persistence of risky sexual behaviors. Lessons from earlier research show that there is still a lack of understanding about HIV/AIDS among fishers and that crucial steps must be taken to strengthen HIV education among fishers to prevent HIV transmission in fishing communities, including Elmina. People who have no/low knowledge of HIV are more likely to disregard primary prevention methods and participate in risky sexual behavior that exposes them to HIV infection [39, 40]. The Komenda-Eguafo-Edina-Abirem (KEEA) Municipality, with Elmina as its capital, bears the highest burden of incidence and prevalence of HIV in the Central Region of Ghana. In 2024, the Municipality recorded 106 new HIV infections across all age groups and 2,407 individuals living with HIV [13].

Research highlights the importance of multiple sources of HIV-related information in improving HIV awareness [48]. Media, healthcare facilities, community outreach programs, peer education, and religious institutions all play crucial roles in disseminating information about HIV [8, 9, 34]. However, researchers have not extensively studied the fishers’ reliance on these sources and their trust in the information they receive. This study seeks to address this knowledge gap by examining the specific sources of HIV-related information that fishers in Elmina use and trust the most.

Although evidence reflects that fishers have poor knowledge of HIV and are a high-risk population group prone to HIV infection, there is a dearth of knowledge regarding their level of knowledge and sources of knowledge in fishing communities in Ghana. Given that an estimated 3 million persons in Ghana are engaged in the fisheries and aquaculture industry [11], a study of fishers’ knowledge of HIV infection prevention strategies and informational resources could be a useful resource for policymaking, research, local-level HIV education, and other key stakeholders in the country’s fishing communities. By assessing the extent of comprehensive HIV knowledge and identifying the most effective information sources, this study will provide valuable insights for designing tailored interventions to improve HIV awareness among fishers. Therefore, the focus of this study is to examine fishers’ awareness and comprehensive knowledge of HIV/AIDS, as well as their knowledge of HIV/AIDS prevention methods. The study investigated fishers’ main sources of knowledge of HIV/AIDS.

## Theoretical foundation

This study is grounded in the Health Belief Model (HBM), which serves as a guiding framework for understanding health-related behaviors and decision-making. HBM, first developed by Rosenstock in 1974, is widely used in public health research. It posits that individuals’ engagement in health-promoting behaviors is influenced by perceptions of susceptibility to a health condition, the severity of that condition, the benefits of preventive measures, the barriers to taking those measures, cues to action, and self-efficacy (Champion & Skinner, 2008).

The HBM offers a structured approach to interpret the awareness of HIV/AIDS, comprehensive knowledge of HIV/AIDS, knowledge of HIV/AIDS prevention methods, and sources of information about HIV/AIDS among fishers in Elmina. Since fishers are considered a group at high risk with little understanding of HIV, this model helps to explore their accurate knowledge and misconceptions about HIV/AIDS, its transmission and measures to prevent HIV infection. This is particularly pertinent, given that prior research indicates that some fishers hold misconceptions about HIV transmission [25, 50].

Additionally, HBM emphasizes self-efficacy, which is a critical factor in determining whether individuals engage in preventive measures, such as consistent condom use or seeking voluntary counseling and testing (VCT) services. Given that many fishers have limited educational backgrounds [29], this emphasis on self-efficacy helps illuminate how literacy levels may affect their ability to engage in preventive behaviors.

Despite its strengths, the HBM has been criticized for its individualistic focus, which may overlook social, economic, and cultural factors that influence fishers’ health decisions. In fishing communities, social norms, peer influences, and economic pressures often have a stronger impact on behavior [27]. Furthermore, the model assumes rational decision-making based on risk assessment; however, studies have found that many fishers act primarily on immediate financial incentives rather than long-term considerations [12].

Moreover, HBM does not explicitly address structural barriers, such as limited access to healthcare in coastal communities, which can significantly hinder fishers’ ability to act on their health beliefs. Recognizing these limitations, this study acknowledges the need to complement HBM with insights from socio-ecological theories, which emphasise broader environmental and societal influences on health behavior.

In conclusion, while the HBM provides a valuable framework for understanding awareness and comprehensive knowledge of HIV/AIDS, as well as HIV/AIDS prevention methods among fishers in Elmina, it is important to consider contextual factors. This necessitates a broader interpretive approach that integrates structural and social determinants of health. In this study, the HBM was employed to guide the interpretation of findings.

## Methods

### Study setting

This study was conducted in the KEEA Municipality, specifically at Elmina. Elmina is situated on the southern coastline of Ghana, bordered to the south by the Gulf of Guinea [15]. Elmina is a historical town due to engagement with European merchants during the colonial era [18]. However, its shoreline is experiencing erosion, which could adversely impact fishing activities [18]. The livelihoods of most Elmina residents are anchored in artisanal fisheries, where fishing, trading, and processing represent the community’s main occupational base [3]. Approximately 75% of Elmina’s residents derive their livelihoods from fishing and associated activities [1].

### Study design

The current study adopted a convergent parallel mixed-methods approach to explore the diverse sources of HIV-related information accessed by fishers. The study was carried out among artisanal marine fishers aged 18 years or older in the Elmina fishing community. The convergent parallel mixed-methods approach facilitates the concurrent collection of quantitative and qualitative data. This method enables the analysis of these data by comparing or relating their findings, thereby enriching the understanding of participants’ experiences [43].

### Sampling strategy

Cochran’s sample size formula was used to estimate the required sample size for the quantitative data, as the target population size could not be specified.

$$\:n=\frac{{Z}^{2}*p\left(1-p\right)\:}{{e}^{2}}$$

Where n = sample size, Z-score = 1.96 for 95% confidence level, *P* = 0., and e = 0.05. Cochran’s formula indicated a required sample size of 384.16, which was rounded to 385. The second author, in partnership with the KEEA Municipality and opinion leaders, identified 10 fisher associations in Elmina and prepared membership lists for each. Probability proportional to size sampling was subsequently employed to distribute the sample across associations using the formula below:

$$\begin{aligned}\mathrm{Fisher}\;\mathrm{Association}\;\left(\mathrm y\right)&={\textstyle\textstyle\frac{\mathrm n\;\left(\mathrm{sample}\;\mathrm{size}\right)}{\mathrm N\;\left(\mathrm{total}\;\mathrm{population}\right)}}\\&\times\mathrm N1\;\left(\mathrm{members}\;\mathrm{in}\right. \\& \left.\mathrm{each}\;\mathrm{association}\right)\end{aligned}$$

To achieve random selection, a lottery system was used to draw 385 fishers from the total membership of 690 across the 10 associations. The allocation of respondents per association is shown in Table 1. Detailed information about the calculation of the sample size and the sampling procedure is found in a previous study [27].

**Table 1:** Sample size per fishers’ association

Name of Association	Population	Sample Size Estimated
Ayisa Fishmongers Association	100	56
Boys’ Hustlers’ Group	90	50
Canoe Fishers Association	80	44
Efar Batafo Fishers Association	80	45
Elmina Canoe Owners Association	60	33
Ghana Co-operative Fisheries Association	56	30
Girls Fish Porters Group	74	41
Konkohin Fishers Association	50	28
Mpobeng Fish Traders Association	40	24
Novisi Fish Mongers Association	60	34
Total	**690**	**385**

The qualitative data for the study involved key informant interviews (KIIs) and focus group discussions (FGDs). For the KIIs, 30 key informants were selected through purposive sampling based on their recognized understanding of fishers’ livelihood strategies and occupational activities in Elmina. The key informants for the study included the KEEA Assembly officials, Directors of other governmental agencies, and community members, including fishers and opinion leaders. Interviews were conducted with four officials from the KEEA Assembly, six Directors of other governmental agencies, and twenty community members.

The FGDs comprised fishers and were stratified by sex, thereby capturing perspectives from both male and female participants. This methodological approach aligns with best practices in qualitative research, which emphasize the importance of considering sex dynamics in data collection [24]. One FGD comprised male fishers, while another comprised female fishers, with each group consisting of ten participants. Participants for the FGDs were identified and selected by the research team using convenience sampling. FGDs were employed to elicit participants’ beliefs and attitudes, while simultaneously enabling the generation and clarification of diverse perspectives [20].

### Measurement of variables

Awareness of HIV/AIDS was measured by asking respondents whether they had heard of HIV/AIDS. The responses were ‘yes’ and ‘no’. Those who indicated yes were classified as ‘have heard of HIV/AIDS,’ while those who indicated no were classified as ‘not heard of HIV/AIDS.’ The measurement of HIV/AIDS awareness in this study is consistent with the measurement used in previous studies [42, 51]. 

Knowledge about HIV/AIDS prevention was measured by asking whether respondents were knowledgeable about HIV/AIDS prevention. The responses were ‘there are ways to prevent HIV/AIDS,’ ‘there are no ways to prevent HIV/AIDS,’ and ‘uncertain.’

Comprehensive knowledge of HIV/AIDS: A person with comprehensive knowledge of HIV/AIDS will be aware that using condoms consistently during sexual activity can reduce the risk of infection and that having just one faithful partner who is HIV-free can also reduce the risk of contracting the disease, that HIV can infect someone who appears healthy, and aware of the two local myths about HIV transmission, that HIV/AIDS can be spread through mosquito bites and supernatural means are false [14]. 

Comprehensive knowledge of HIV/AIDS was computed as a composite indicator by using the following five questions: (a) can a healthy-looking person have HIV? (b) can you become infected with HIV/AIDS by having sexual intercourse without a condom? (c) can you become infected with HIV/AIDS from mosquito bites? (d) can you become infected with HIV/AIDS through witchcraft? and (e) can the risk of HIV infection be reduced by having sexual intercourse with only one faithful, uninfected partner? A correct response is assigned a value of 1, while a wrong response is assigned a value of 0. Therefore, the score ranges from 0 to 5. The mean and median scores were computed. As shown in Table 2, the mean and median scores were 3.3 and 4.0, respectively. The median score of 4 was used as the threshold to classify comprehensive knowledge of HIV/AIDS as ‘high’ or ‘low.’ Any score below 4 (the median score) was considered as low comprehensive knowledge of HIV/AIDS, while a score of 4 or higher was considered as high comprehensive knowledge of HIV/AIDS.

**Table 2:** Computation of comprehensive knowledge of HIV/AIDS

Number of indicators answered	Frequency	Percent
Could not answer any (0) of the indicators correctly	12	3.1
Could answer one (1) of the indicators correctly	12	3.1
Could answer two (2) of the indicators correctly	40	10.4
Could answer three (3) of the indicators correctly	107	27.8
Could answer four (4) of the indicators correctly	199	51.7
Could answer all five (5) indicators correctly	15	3.9
Total	385	100.0
Mean		3.3
Median		4.0
Computed Comprehensive Knowledge		
Low	171	44.4
High	214	55.6

Source: Field survey, July 2017

This study also examined the socio-demographic characteristics of respondents. These characteristics included sex (male and female), age (< 25, 25–34, 35–44, 45–54, and 55+), mobility status (mobile fisher and non-mobile fisher), and educational level (no education, Middle/Junior High School (JHS), and secondary/vocational and higher). Other characteristics included religion (Islam, African Traditionalist, no religion, and Christianity), marital status (never married, cohabiting/informal/consensual, married, divorced/separated/widowed), and type of fishing occupation (fish catch group, post-harvest group, maintenance and repair group, and porters and errand group).

### Data collection

In July and August 2017, data collection was carried out, including a survey of 385 fishers and KIIs and FGDs for the qualitative component of the study. The questionnaire for the survey and the interview guides for the KII and FGD are available in Supplementary Material 1.

Enumerators with at least 2 years of experience in data collection were recruited and trained to assist with quantitative and qualitative data collection. Enumerators were trained on the study’s purpose and on how to solicit information from respondents.

### Data analysis

Descriptive statistics (frequency and percentage) were used to describe the socio-demographic characteristics of fishers for the quantitative data. Also, the chi-square test and Fisher’s exact test were used to examine the association between awareness of HIV/AIDS, knowledge of HIV/AIDS prevention methods, main sources of HIV/AIDS knowledge, and fishers’ socio-demographic characteristics. Fisher’s exact test is the appropriate statistical test for categorical variables when expected cell counts are less than 5, as it provides exact probabilities without relying on large-sample approximations [33]. The Statistical Package for the Social Sciences version 27 was used to analyzed the quantitative data. Associations between variables were considered statistically significant at a 95% confidence interval (*p* < 0.05).

The qualitative data (KIIs and FDGs) were audio-recorded and transcribed to capture participants’ perspectives word-for-word. Thematic analysis was also used to analyze the transcripts in the following stages. At the initial stage, the second author read all transcripts thoroughly to gain a comprehensive understanding of participants’ perspectives. In the second stage, the second author assigned initial codes to participants’ perspectives that related to the research objectives. Afterwards, the second author merged codes that related to similar issues to form initial themes. The second author held a debriefing session with all co-authors, during which initial codes and themes were reviewed, and feedback was used to revise them where necessary. In the last stage, all authors used the themes and their related codes to write the results section of this study. During the data collection and analysis, all decisions made were documented to enhance the study’s trustworthiness. Furthermore, the quantitative and qualitative findings were triangulated where applicable to provide a nuanced understanding of participants’ experiences.

### Ethical issues

The University of Ghana’s Ethics Committee for the Humanities (# ECH 118/16–17) reviewed and approved the study. Written informed consent was secured from each participant, and all procedures complied with the ethical standards of the Declaration of Helsinki.

## Results

### Socio-demographic characteristics of fishers

Among the 385 respondents interviewed for the study, there were more female fishers (51.4%) than male fishers (48.6%). Also, 53.5% of respondents were aged 35 or older, while 54.3% were mobile fishers (Table 3). Most respondents had attained formal education (66.5%) and were Christians (82.1%). Also, 55.6% of respondents were either married or living with a partner. Furthermore, 38.7% of respondents were engaged in post-harvest fishing activities.

**Table 3:** Socio-demographic characteristics of respondents

Socio-demographic characteristics	Frequency	Percent
Sex		
Male	187	48.6
Female	198	51.4
Age		
< 25	86	22.3
25–34	93	24.2
35–44	115	29.9
45–54	50	13.0
55+	41	10.6
Mobility status		
Mobile Fisher	209	54.3
Non-Mobile Fisher	176	45.7
Educational level		
No education	129	33.5
Middle/JHS	207	53.8
Secondary/vocational and higher	49	12.7
Religion		
Islam	21	5.5
African Traditionalist	19	4.9
No religion	29	7.5
Christianity	316	82.1
Marital status		
Never married	112	29.1
Cohabiting/Informal/Consensual	34	8.8
Married	180	46.8
Divorced/separated/widowed	59	15.3
Type of fishing occupation		
Fish Catch Group	98	25.5
Post-harvest Group	149	38.7
Maintenance and Repair Group	55	14.3
Porters and Errand Group	83	21.6

Source: Fieldwork, July-August 2017

### Awareness of HIV/AIDS

The percentage distribution of the respondents who have heard of HIV/AIDS is shown in Table 4. The majority (97.9%) of the respondents were aware of HIV/AIDS, while 2.1% indicated that they were not aware of HIV/AIDS. Also, there was no significant association between HIV/AIDS awareness and the socio-demographic characteristics of respondents (*p* = 0.527, 0.584, 0.331, 0.550, 0.766, 0.062, 0.620).

**Table 4:** Percentage of respondents who have heard of HIV/AIDS (awareness of HIV/AIDS) by socio-demographic characteristics

Socio-demographic characteristics	Have you heard of HIV/AIDS? (Yes)		
	Frequency	Percent	*P*-value
Sex			0.725^
Male	184	98.4	
Female	193	97.5	
Age			0.344^
< 25	86	100	
25–34	91	97.8	
35–44	112	97.4	
45–54	49	98.0	
55+	39	95.1	
Mobility status			0.477^
Mobile	206	98.6	
Non-mobile	170	97.1	
Educational level			0.785^
No Education	126	97.7	
Middle/JHS education	202	97.6	
Secondary/vocational and higher	49	100.0	
Religion			0.799^
Islam	21	100.0	
African Traditionalist	19	100.0	
No religion	28	96.6	
Christianity	309	97.8	
Marital status			0.210^
Never married	109	97.3	
Cohabiting/Informal/Consensual	34	100	
Currently married	177	98.3	
Divorced/ Separated/Widowed	57	98.3	
Type of fishing occupation			0.602^
Fish Catch Group	97	99.0	
Post-harvest Group	145	97.3	
Maintenance and Repair Group	53	96.4	
Porters and Errand Group	82	98.8	
Total	**377**	**97.9**	

^ = Fisher’s exact test

Source: Field survey, July 2017

### Comprehensive knowledge of HIV/AIDS

This study also assesses respondents’ comprehensive knowledge of HIV/AIDS. Comprehensive HIV/AIDS knowledge was assessed using five indicators: awareness that condom non-use increases HIV infection risk; rejection of the misconception that healthy-looking individuals cannot carry HIV; rejection of the misconception that mosquitoes can transmit HIV; rejection of the misconception that HIV can be transmitted through witchcraft; and acknowledgement that HIV risk is reduced through fidelity to one uninfected partner. The result in Table 2 indicates that 55.6% of the respondents have a high comprehensive knowledge of HIV/AIDS, while 44.4% had low comprehensive knowledge of HIV/AIDS.

Among the indicators of comprehensive HIV/AIDS knowledge, 92.2% of respondents recognized unprotected sex as a transmission route of HIV, while 77.1% acknowledged that healthy-appearing individuals may be HIV infected (Table 5). However, only 1.5% of respondents reported reduced risk of HIV through fidelity to one uninfected partner, while 7.3% and 4.9% incorrectly attributed HIV infection to witchcraft and mosquito bites, respectively.

**Table 5:** Comprehensive knowledge of HIV/AIDS indicators

Indicators	Yes *n* (%)	No *n* (%)	Don’t know *n* (%)
You can be infected with HIV/AIDS by having sexual intercourse without a condom	355 (92.2)	10 (2.6)	20 (5.2)
A healthy-looking person can have HIV	297 (77.1)	56 (14.5)	32 (8.4)
You can be infected with HIV/AIDS through mosquito bites	19 (4.9)	331 (86.0)	35 (9.1)
You can be infected with HIV/AIDS through witchcraft	28 (7.3)	337 (87.5)	20 (5.2)
HIV risk is reduced by having sexual intercourse with only one faithful uninfected partner	5 (1.3)	301 (78.2)	79 (20.5)

Source: Field survey, July 2017

The qualitative findings supported the quantitative findings that some fishers have some misconceptions about HIV transmission. During the FGDs, some fishers narrated that HIV could be acquired supernaturally, particularly through witchcraft.

*Some of the ladies here are witches*,* and others have evil spirits*,* so when you have intercourse with them*,* they transmit HIV into your body. As a result*,* many young men and women in Elmina have contracted the virus.* [FGD 2, Male Fisher]

Also, some participants explained that they could not acquire HIV because they believed sweating and exposure to sea water would kill the virus. Consequently, they perceived themselves as not at risk of HIV infection.

*Because HIV germs are killed by salty sea water*,* fishers do not acquire AIDS. Because we bathe in the sea*,* we work so hard maintaining our boats and canoes*,* and fishermen tend to sweat a lot profusely. HIV is destroyed when we sweat.* [FGD 1, Male Fisher]

The misconceptions about HIV/AIDS among participants are expected, given that many fishers avoid HIV education programs.

*When we organize community durbars*,* the fisher folks are usually our target audience. Incidentally*,* they don’t show up to participate. Most of them are not interested*,* and the others who show up sit in for a few minutes and leave to go back to fish*,* process fish*,* or mend their nets for the next fishing expedition. It is very worrying to run a program and not be able to engage your target audience*. (KII 4, NGO Representative)

Furthermore, some fishers regard the content of HIV education programs as frightening and blame-centered, which discourages engagement and reinforces misconceptions. Additionally, the Information, Education, and Communication materials for the HIV education programs are produced in English, which some fishers cannot understand due to limited English literacy. An opinion leader had this to say:

*The programs are too dry. All they do is talk and frighten us with the disease. The way they describe the disease makes it look as if it is your fault to get the disease. This is why most people here don’t show interest and would prefer to either listen to the radio or watch HIV programs on TV. I believe they should include role-plays*,* sketches*,* and documentaries that relate to the everyday lives of fisherfolks. That is how to make people feel that the disease affects ordinary people like us*. (KII 3, Opinion leader)

Moreover, key informants observed that HIV education programs were sporadic, largely due to financial constraints, which compromised their implementation. The lack of consistency in HIV education programs undermines fishers’ knowledge of HIV/AIDS.

*HIV/AIDS awareness programs cover not only fishers but the general population within Elmina Township. However*,* I must admit it has not been consistent. We only have it when we have funds.* (KII 13, Municipal Health Directorate)

### Knowledge of HIV/AIDS prevention

**Fig. 1:**
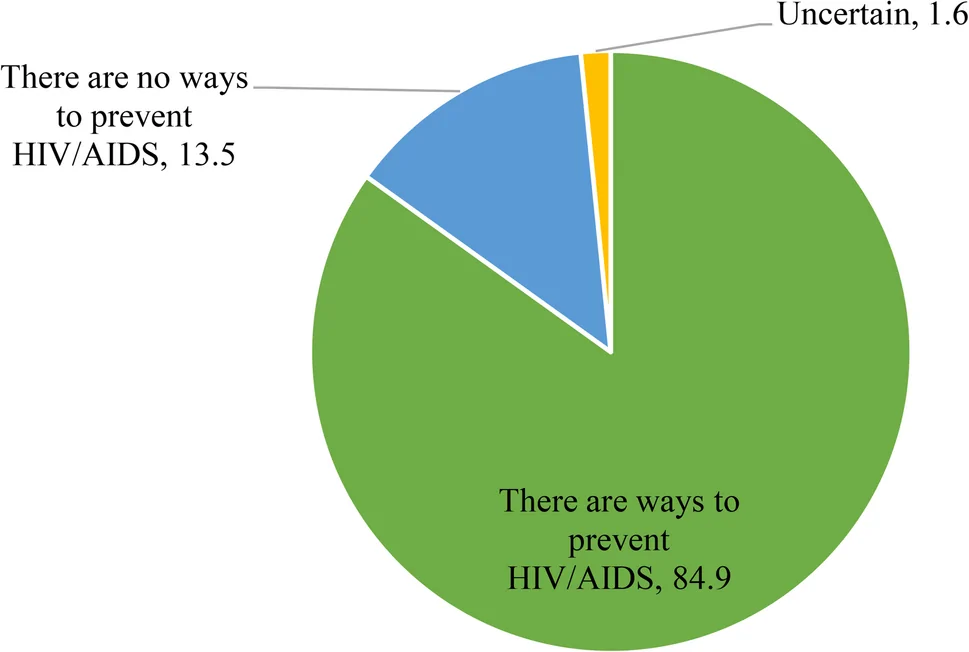
Respondents’ knowledge of HIV/AIDS prevention

Respondents were asked about their knowledge of HIV/AIDS prevention. From Fig. 1, most (84.9%) respondents reported that there are ways to prevent HIV/AIDS, while 13.5% reported that there are no ways to prevent HIV/AIDS. Also, 1.6% of respondents were uncertain whether HIV/AIDS could be prevented or not.

Furthermore, respondents who indicated that there are ways to prevent HIV/AIDS were asked what methods can be used to prevent HIV/AIDS infection. Table 6 provides results on respondents’ knowledge of HIV/AIDS prevention methods by age, sex, mobility status, and educational level.

**Table 6:** Knowledge of HIV/AIDS prevention methods by respondents’ sex, age, mobility status, and educational level

Background Characteristics	Abstinence	Being faithful	Avoiding multiple/casual partners	Using condoms	Use of IUD	Use of contraceptive pills	Avoid blades and other sharp objects	Avoid direct contact with infected persons	*P*-value
Sex									< 0.001^
Male	11.2	17.1	4.3	59.9	3.2	1.1	2.1	1.1	
Female	19.7	33.8	2.0	34.8	3.0	4.0	0.0	2.5	
Age									< 0.001^
< 25	11.6	16.3	4.7	59.3	2.3	2.3	1.2	2.3	
25–34	9.7	28.0	1.1	52.7	2.2	2.2	2.2	2.2	
35–44	9.6	26.1	1.7	52.2	1.7	5.2	0.9	2.6	
45–54	36.0	22.0	10.0	32.0	0.0	0.0	0.0	0.0	
55+	29.3	43.9	0.0	12.2	14.6	0.0	0.0	0.0	
Mobility status									< 0.001^
Mobile fisher	9.6	20.1	4.8	57.4	3.3	1.9	1.0	1.9	
Non-Mobile fisher	22.9	32.0	1.1	34.9	2.9	3.4	1.1	1.7	
Educational level									< 0.001^
No Education	18.6	18.6	3.9	40.3	8.5	4.7	0.8	4.7	
Middle/JHS education	15.0	30.0	3.4	48.8	0.5	1.0	1.0	0.5	
Secondary/vocational and higher	10.2	26.5	0.0	57.1	0.0	4.1	2.0	0.0	
Total	**15.6**	**25.7**	**3.1**	**47.0**	**3.1**	**2.6**	**1.0**	**1.8**	

^ = Fisher’s exact test

Source: Field survey, July 2017

From Table 6, condom use, being faithful, and abstinence emerged as the main HIV/AIDS prevention methods known by respondents. Most respondents (47.0%) reported condom use as an HIV/AIDS prevention method, while a quarter of respondents (25.7%) reported being faithful as an HIV/AIDS prevention method. Also, 15.6% of respondents reported abstinence as an HIV/AIDS prevention method. Approximately 3.0% of respondents reported the use of intrauterine devices (IUDs) as an HIV prevention method, while 2.6% reported the use of contraceptive pills as an HIV prevention method. Avoiding blades and other sharp objects was identified as an HIV prevention method by 1.0% of respondents.

Male respondents (59.9%), those younger than 25 years (59.3%), mobile fishers (57.4%), and respondents with Middle/JHS education (48.8%) significantly reported higher proportions of condom use as an HIV/AIDS prevention method (*p* < 0.001) (Table 6). Also, female respondents (33.8%), those aged 55 years and older (43.9%), non-mobile fishers, and respondents with Middle/JHS education (30.0%) reported significantly higher proportions of being faithful as an HIV/AIDS prevention method (*p* < 0.001). Similarly, female respondents (19.7%), those aged 45–54 (36.0%), non-mobile fishers (22.9%), and respondents with no formal education (18.6%) significantly reported higher proportions of abstinence from sexual intercourse as an HIV/AIDS prevention method (*p* < 0.001).

Among the three main HIV/AIDS prevention methods, a higher proportion of older fishers (aged 45 years and older) generally identified being faithful and abstinence as HIV/AIDS prevention methods (Table 6). This quantitative finding supports the qualitative finding that older adult fishers, who are typically immobile, have more time to participate in HIV/AIDS programs at fish landing sites. In other words, a greater proportion of them had the chance to learn more about HIV/AIDS than fishers of other age groups that are highly mobile and rarely have similar opportunities to attend and participate in HIV/AIDS education programs. An older adult female who participated in the female FGD reported the following:

*Every time the health officers visit*,* I participate in HIV/AIDS public education programs. It is helpful because I can listen to the program officials and seek clarification to gain more knowledge about the virus and disease. I also get to hear about other people’s experiences with HIV/AIDS-related concerns.* [FGD 3, Female Fisher]

Another participant explained that the venue for HIV/AIDS programs coincided with the same location where older fishers’ gatherings were held. Consequently, this facilitated their participation in HIV/AIDS programs.

*The Ayisa market is the main venue for most education programs and is where fish traders sit to process their fish. HIV/AIDS programs are held at the Ayisa market because it is a convenient point in the fishing community where many fishers can participate. Incidentally*,* the fish market is the usual gathering place for older adult fishers*,* either for processing fish or for waiting for fish supplies from new expeditions; hence*,* they have the opportunity to participate in HIV/AIDS programs.* [KII 1, NGO Representative]

Furthermore, Table 6 shows that non-mobile fishers generally have more knowledge of HIV/AIDS prevention methods than mobile fishers. For instance, non-mobile fishers were more knowledgeable about being faithful (32.0%), abstinence from sexual intercourse (22.9%), and avoiding blades and other sharp objects (1.0%) as HIV/AIDS prevention methods compared to mobile fishers. This is largely because non-mobile respondents have more access to information on HIV/AIDS prevention strategies than mobile respondents. The qualitative finding also supported this quantitative finding. Participants indicated that immobile fishers often participate in their HIV/AIDS prevention programs more than mobile fishers since they are often available, which tends to increase their knowledge on HIV/AIDS and its prevention methods.

*Many of the fishers who take part in our programs are primarily immobile. Typically*,* they are made up of women*,* older adult males*,* and other fishers whose job is restricted to the landing sites. They have more time to take part in HIV/AIDS intervention programs than their mobile colleagues*,* who virtually never have time for such programs.* [KII 2, Municipal Health Officer]

### Sources of information about HIV/AIDS

To aid the organization of health promotion programs and make critical decisions about how to reach individuals with HIV/AIDS knowledge and intervention activities, it is crucial to understand fishers’ sources of information about HIV/AIDS. Table 7 shows respondents’ main sources of knowledge about HIV/AIDS.

**Table 7:** Main sources of knowledge of HIV/AIDS information by respondents’ sex, age, mobility status, and educational level

Background Characteristics	Television	Radio	Publications (Newspapers, Pamphlets, Posters)	Health care workers	Town hall/ community durbars	Internet	*P*-value
Sex							< 0.001^^
Male	10.7	54.5	18.7	1.6	4.3	10.2	
Female	26.8	36.9	3.5	11.6	10.6	10.6	
Age							< 0.001^
< 25	11.6	44.2	10.5	3.5	4.7	25.6	
25–34	19.4	36.6	17.2	9.7	4.3	12.9	
35–44	27.0	48.7	9.6	4.3	7.8	2.6	
45–54	16.0	58.0	4.0	10.0	8.0	4.0	
55+	14.6	43.9	9.8	9.8	19.5	2.4	
Mobility status							0.450^
Mobile fisher	18.7	49.8	10.0	5.3	6.2	10.0	
Non-Mobile fisher	19.4	40.6	12.0	8.6	9.1	10.3	
Educational level							< 0.001^
No Education	24.0	58.1	1.6	7.8	8.5	0.0	
Middle/JHS education	17.9	44.0	10.6	6.8	8.2	12.6	
Secondary/vocational and higher	10.2	18.4	36.7	4.1	2.0	28.6	
Total	**19.0**	**45.5**	**10.9**	**6.8**	**7.5**	**10.4**	

^ = Fisher’s exact test; ^^ = Chi square test

Source: Field survey, July 2017

From Table 7, the two primary sources of information about HIV/AIDS were radio (45.5%) and television (19.0%). Healthcare workers (6.8%) were the least reported main source of information about HIV/AIDS by respondents. In comparison, one-tenth (10.4%) of respondents reported the internet as a main source of information about HIV/AIDS.

Table 7 further indicates sex differentials in the main sources of information about HIV/AIDS (*p* < 0.001). For instance, more male respondents reported the radio (54.5%) and publications (18.7%) as the main sources of information about HIV/AIDS, while more female respondents reported television (26.8%), health care workers (11.6%), townhall/community durbars (10.6%) and internet (10.6%) as the main sources of information about HIV/AIDS.

Also, respondents’ age was significantly associated with the main sources of information about HIV/AIDS (*p* < 0.001) (Table 7). Specifically, more respondents aged 45–54 (58.0%) reported the radio as their main source of HIV/AIDS information, while most respondents aged 35–44 (27.0%) reported the television as their main source of HIV/AIDS information. In addition, most respondents aged less than 25 (25.6%) reported the internet as the main source of information about HIV/AIDS.

Furthermore, educational level was significantly associated with the main sources of information about HIV/AIDS (*p* < 0.001) (Table 7). Most respondents with no formal education reported radio (58.1%), television (24.0%), health care workers (7.8%) and town hall/community durbars (8.5%) as the main sources of information about HIV/AIDS. Additionally, more respondents with secondary/vocational and higher education reported publications (36.7%) and the internet (28.6%) as the main sources of information about HIV/AIDS.

## Discussion

This study examined fishers’ awareness and comprehensive knowledge of HIV/AIDS. It also investigated knowledge about HIV/AIDS prevention methods as well as sources of information about HIV/AIDS. This study found that although 97.9% of fishers have ever heard about HIV/AIDS, a significant percentage of them (44.4%) demonstrated low comprehensive knowledge of the disease. Fishers’ high prevalence of awareness of HIV/AIDS could be attributed to HIV/AIDS education by governmental and non-governmental organizations in the study area [29]. Also, the prevalence of comprehensive HIV/AIDS knowledge among respondents (55.6%) in this study was higher than that reported by Kwagonza et al., [25] in Uganda (51.0%). Whereas their study examined adults in a fishing community, ours focused exclusively on fishers, which may account for the observed differences.

FGDs with participants in our study also revealed gaps in fishers’ knowledge about HIV/AIDS transmission. Some participants attributed HIV transmission to supernatural causes, while others believed that regular contact with seawater during fishing activities protected them from HIV infection. These findings corroborate earlier studies that identified low risk perception of HIV infection and persistent misconceptions regarding HIV transmission among fishers [25, 50]. The HBM posits that awareness and knowledge shape individuals’ perceptions of susceptibility and severity regarding health conditions. Accordingly, the awareness and limited knowledge of fishers about HIV/AIDS may influence their perceived risk of contracting the disease and its potential consequences. These findings therefore align with the HBM construct of perceived susceptibility.

Some participants reported that HIV education programs were perceived as frightening and blame-centered, while the accompanying IEC materials were produced in English. These factors have important implications for the overall effectiveness of HIV education initiatives. These findings are concerning, as fishers face elevated vulnerability to HIV infection due to behavioral risk factors such as unprotected sexual activity, transactional sex, multiple partners, and excessive alcohol use [27, 31, 35]. The findings highlight the need for governmental and non-governmental organizations to strengthen HIV/AIDS education in the study area by ensuring that programs are culturally appropriate, non-stigmatizing, and delivered in local languages. Such improvements would enhance fishers’ comprehensive knowledge of HIV/AIDS and increase the effectiveness of education initiatives. Also, this study emphasizes the importance of long-term funding to maintain regular HIV/AIDS education initiatives that improve fishers’ understanding of the disease.

Regarding knowledge of fishers on HIV/AIDS preventive measures, it was found that 84.9% of respondents believed there were ways to avoid contracting AIDS. In comparison, 13.5% disagreed, and 1.6% were unsure. Also, most fishers knew that using condoms (47.0%), restricting sexual intercourse to one uninfected partner (25.7%), and abstaining from sexual activity (15.6%) can prevent HIV/AIDS infection. In terms of sex, more male fishers reported condom use and avoiding multiple/casual partners as HIV/AIDS prevention methods. In contrast, more female fishers reported abstinence and fidelity as HIV prevention methods.

The sex-based differences observed in fishers’ knowledge of HIV/AIDS prevention methods highlight the influence of gendered socialization and access to information. Male fishers’ greater recognition of condom use and of avoiding multiple/casual partners as prevention strategies reflects broader patterns in which men are more frequently exposed to biomedical and behavioral prevention messaging, often through peer networks and occupational mobility [30, 46]. In contrast, female fishers’ stronger association of abstinence and fidelity with HIV prevention is consistent with cultural expectations that frame women’s sexual health knowledge around moral and relational norms, while simultaneously limiting their exposure to condom-related education due to stigma and reduced negotiation power [16, 17]. Addressing these disparities requires gender-responsive education strategies that expand women’s knowledge of biomedical prevention methods while challenging restrictive norms that confine their understanding of HIV risk reduction [2]. Within the HBM framework, understanding preventive measures may enhance the perceived benefits of actions, as fishers acknowledge the efficacy of specific behaviors (such as condom use and abstinence) in mitigating their risk. However, a previous study by Kyei-Gyamfi [30] found low condom use among fishers in the study area.

The study found that only 1.0% of respondents reported avoiding blades and other sharp objects as an HIV prevention measure. Since fishers, particularly fishmongers, routinely use knives and scaling tools in their daily work, this finding has implications for their protective practices. In relation to the HBM, the low recognition of sharp-object avoidance as a preventive measure may reduce motivation to adopt such behavior, as fishers do not perceive themselves to be susceptible to HIV/AIDS through the use of these tools.

Furthermore, 5.7% of respondents held the misconception that IUDs and contraceptive pills could prevent HIV. This is concerning, as such beliefs may lead to inappropriate reliance on these protection methods. The finding underscores the urgent need to sensitize fishers about HIV transmission and effective prevention strategies in order to correct these misconceptions.

Additionally, this study found that non-mobile fishers were generally more knowledgeable about HIV/AIDS prevention methods than mobile fishers since non-mobile fishers had a greater opportunity to participate in HIV/AIDS programs due to their availability than mobile fishers. Mobile fishers’ mobility limits their access to health education, potentially restricting their ability to adopt preventive behaviors, as shown in previous research [28, 29]. Restricted access to health education for mobile fishers may result in diminished perceived benefits and self-efficacy, both essential components of the HBM that are vital for facilitating behavior change.

Furthermore, older fishers were generally more knowledgeable about HIV/AIDS prevention methods than younger fishers. Older fishers participated in more HIV/AIDS education programs than younger fishers, largely because their workplaces were closer to the venues for these programs, and they were more available. Younger fishers, being frequently at sea, had fewer opportunities to be exposed to HIV/AIDS education programs. As a result, older fishers gained greater exposure to HIV/AIDS education programs, which shaped their knowledge of HIV/AIDS prevention. From an HBM perspective, continuous exposure to health information may strengthen perceived susceptibility, perceived severity, and self-efficacy, thereby encouraging protective health behaviors among older fishers. It must be acknowledged that fishers exposed to HIV/AIDS education, especially its prevention methods, may influence their confidence in their ability to protect themselves from HIV/AIDS, which can affect their self-efficacy, as outlined in HBM.

This study found radio and television as the main sources of knowledge about HIV/AIDS among fishers. Other sources of knowledge about HIV/AIDS among fishers were publications (newspapers, pamphlets, posters, etc.), internet, town hall/community durbars, and health care workers. Our study found that more female fishers identified television as their main source of HIV/AIDS knowledge compared to males. This outcome is expected, as women generally spend more time watching television, thereby increasing their exposure to HIV/AIDS information [5, 41]. Moreover, this study found that male fishers identified radio as their main source of HIV/AIDS knowledge compared to females. This aligns with previous studies documenting that men generally listen to radio more frequently than women [4, 36].

The findings indicate that a higher proportion of females, younger respondents (those aged below 35 years), and those with secondary/vocational or higher education identified the internet as their main source of HIV/AIDS knowledge. This pattern reflects broader demographic trends in digital engagement. Prior studies consistently show that females use the internet more frequently than males [44, 47], and such exposure increases their access to HIV/AIDS information, thereby enhancing their knowledge of HIV/AIDS. Younger persons are generally more technologically savvy and thus more likely to seek health information online [32], which may increase their access to HIV/AIDS education. Also, educational attainment facilitates internet use [7, 21], and therefore, respondents with secondary/vocational and higher education may have greater digital literacy and capacity to navigate online resources compared to those with limited education, which can increase their exposure to HIV/AIDS information. These findings highlight the need to develop interventions that address disparities in digital access to promote access to HIV/AIDS education.

The diverse information sources act as cues to action, a critical element in the HBM, encouraging fishers to adopt or sustain preventive behaviors against HIV/AIDS. These findings support previous studies that found radio, television, the internet, and healthcare workers as sources of knowledge about HIV/AIDS [6, 37, 49].

### Limitations and strengths

The following study limitations were noted and are duly acknowledged. Firstly, the cross-sectional design of the study limits the ability to draw causal inferences about the relationships between awareness of HIV/AIDS and the socio-demographic characteristics of fishers, and between the main sources of knowledge of HIV information and the socio-demographic characteristics of fishers. Future studies should employ a longitudinal research design to provide a clearer picture of these associations. Secondly, the reliance on self-reported data introduces the potential for recall and social desirability bias, especially given the sensitive nature of HIV-related topics. Respondents may have either overstated or understated their HIV/AIDS awareness and knowledge.

Additionally, the study was conducted within the Elmina fishing community. Hence, the findings of this study may not be generalizable to all fishers in Ghana. Variations in cultural, economic, and educational contexts across different fishing communities suggest the need for multi-site studies to improve external validity. Furthermore, the study was conducted in 2017, with findings published in 2026. Although changes in HIV/AIDS knowledge among fishers may have occurred during this nine-year interval, the results remain relevant for strengthening HIV/AIDS sensitization efforts in Elmina.

Despite its limitations, the study possesses several key strengths. Employing a convergent parallel mixed-method design, integrating quantitative survey data with qualitative perspectives, allows for a holistic understanding of fishers’ HIV/AIDS knowledge and prevention methods. This methodological triangulation strengthens the credibility of the findings and offers deeper contextual insights.

Furthermore, the study’s focus on fishers, a high-risk yet frequently neglected population, highlights its relevance for shaping effective public health interventions. The findings of this study provide evidence to support tailored policy and programmatic responses.

### Policy and practice implications

This study’s findings identify critical areas for policy intervention and practical strategies to enhance HIV/AIDS awareness and prevention among fishers in Elmina. Considering the mobility and high-risk nature of fishers, policymakers and program designers need to create targeted, accessible, and sustainable interventions to address the gaps in HIV/AIDS knowledge and prevention methods. Policymakers should prioritize integrating mobile-friendly HIV/AIDS education initiatives to effectively address the distinct mobility challenges faced by fishers, especially mobile fishers. Given the limited access of mobile fishers to static educational programs, implementing mobile health (mHealth) interventions, such as SMS-based messaging, interactive voice response systems, and mobile applications, can facilitate the provision of real-time, context-specific HIV/AIDS prevention information. These methods ensure that educational opportunities are accessible to fishers, regardless of their location or work commitments. Additionally, to address disparities in health education among mobile and non-mobile fishers, this study proposes boat-landing outreach programs that extend HIV/AIDS education to mobile fisher populations.

The study highlights the need to use varied communication channels that align with the preferences of distinct demographic groups. Older fishers and male respondents favor radio as an information source, whereas younger fishers show a preference for the internet. Krause’s [23] study in Australia found that most older adults listen to radio. HIV/AIDS prevention programs should leverage these preferences by integrating radio discussions, television broadcasts, and social media campaigns that deliver engaging, factual, and culturally relevant messages on HIV/AIDS prevention. There is a significant necessity to enhance peer education programs in fishing communities. The important role of social interactions in shaping health behaviors suggests that training local fisher leaders and peers as HIV/AIDS education ambassadors may improve knowledge dissemination. Peer education can also enhance fishers’ self-efficacy by providing relatable role models who demonstrate how to adopt preventive behaviors effectively. Community-based interventions must be structured to rectify misconceptions regarding HIV/AIDS, guaranteeing that fishers obtain accurate, evidence-based information from reliable sources.

A significant policy implication is the integration of HIV/AIDS prevention education into vocational training related to fisheries and cooperative associations. Embedding HIV/AIDS awareness modules into fisher training programs will allow policymakers to institutionalize consistent health education, thereby integrating it into fishers’ professional development. Incorporating the principles of the HBM into these educational modules by addressing perceived susceptibility, perceived severity, perceived benefits, perceived barriers, cues to action, and self-efficacy will enhance the likelihood that fishers will internalize and act on HIV/AIDS prevention information. Thus, policymakers and fisher associations should integrate HIV/AIDS education into cooperative meetings, peer-mentorship programs, and vocational training schemes to enhance fishers’ knowledge of HIV/AIDS.

## Conclusion

The study underscores the complex relationship between awareness and comprehensive knowledge of HIV/AIDS and knowledge about HIV/AIDS prevention methods among fishers in Elmina. Although general awareness of HIV/AIDS is high among fishers, there are notable gaps in their knowledge of HIV/AIDS, especially HIV/AIDS prevention methods. For instance, during FGDs, it emerged that there is a perceived belief among fishers that HIV/AIDS can be transmitted through supernatural means. The quantitative data found that 34.2% of fishers do not know that avoiding the sharing of sharp or piercing objects can prevent the transmission of HIV/AIDS. In addition, fishers derived their HIV/AIDS knowledge from diverse sources, including radio, television, and the internet.

Utilizing the HBM, these knowledge gaps indicate that although fishers may possess a general awareness (cue to action), their perceived susceptibility and perceived severity of HIV/AIDS may be low or shaped by misconceptions, which in turn impacts their adoption of preventive behaviors. Enhancing fishers’ perceived benefits of preventive actions, such as consistent condom use and avoiding the sharing of sharp objects, while reducing perceived barriers, including myths about supernatural transmission, are equally essential for improving their preventive practices. The findings of this study highlight the need for policymakers to tailor HIV/AIDS sensitization efforts aimed at fishers to bridge their HIV/AIDS knowledge gap, especially HIV/AIDS prevention methods and myths about HIV transmission (transmission of HIV/AIDS through mosquito bites and supernatural means) to reduce their risk of HIV/AIDS infection. Policymakers could utilize channels, such as radio and television, for their HIV/AIDS sensitization efforts for fishers since radio and television are the dominant sources of fishers’ HIV/AIDS knowledge. Future research should employ a national mixed-methods design to examine HIV/AIDS awareness, comprehensive knowledge, prevention knowledge, and sources of information among fishers. A study of this scope would generate a more nuanced and representative understanding of HIV/AIDS knowledge within this high-risk group. The findings of such studies could then be used to develop context-specific interventions tailored to the cultural and occupational realities of fishers, thereby strengthening HIV/AIDS sensitization efforts and reducing fishers’ vulnerability to HIV infection.

## Supplementary Information

Supplementary Material 1.

## Data Availability

The datasets generated and/or analyzed during the current study are available from the corresponding author upon reasonable request.

## Abbreviations

AIDS: Acquired Immunodeficiency Syndrome
FAO: Food and Agriculture Organization
HIV: Human Immunodeficiency Virus
JHS: Junior High School
SPSS: Statistical Package for the Social Sciences
UNAIDS: Joint United Nations Programme on HIV/AIDS
WHO: World Health Organization
