# Supplementary material for: Sailing towards health: HIV/AIDS awareness, comprehensive knowledge, prevention methods, and information sources among fishers in Elmina, Ghana
Source: BMC Public Health. 2026 Aug 17;26:3354. doi: 10.1186/s12889-026-29040-2 (PMC13756915; doi:10.1186/s12889-026-29040-2)
Supplement: Supplementary file 1 [file 12889_2026_29040_MOESM1_ESM.docx]

## QUESTIONNAIRE FOR FISHERS

Thank you for agreeing to be interviewed today, I appreciate it. My name is *Sylvester Kyei-Gyamfi*. I am a PhD student of the Centre for Migration Studies, University of Ghana, Legon – Accra. I am carrying out this field research work as part of academic work for my Doctorate Degree. The research topic is on “*Mobility and HIV Risk among Fishers in Elmina Fishing Community in the Central Region of Ghana’*. This is will be a question and answer session concerning your movement from your work, movements, settlement conditions, knowledge, attitudes and practices concerning HIV. I will be grateful if you allow me to take a few minutes of your time to engage you in this session. You are assured that any information provided will be treated confidential and used solely for academic purposes only. Please answer all questions openly and honestly. Remember there is right or wrong answer. I am interested in your own personal experiences and opinions. If you want to skip any questions you are free to do so but it is really important that you answer as many as possible. If you do not understand any of the questions just let me know and I will try to explain. Thank you.

**IDENTIFICATION**

| ID1 | Questionnaire ID Number:……………….. | Place of interview…………………………… |  |
| --- | --- | --- | --- |
| ID2 | Telephone number:................................ | Whatsapp number………………………… |  |

**SECTION A: SOCIO-DEMOGRAPHIC BACKGROUND**

To start I would like to ask some questions about yourself:

| DB1 | How old are you today? | 1. <25 2. 25-34 3.45-544.55-645.65-74 6.75+99. Don’t Know |  |
| --- | --- | --- | --- |
| DB2 | What is your sex? | 1. Male 2. Female |  |
| DB3 | What is your highest educational level achieved? | 1.Never been to school 2. Primary3. JSS/JHS  4.Middle School 5.SSS/SHS 5. Vocational/ Technical/ Commercial 6.Post-Middle/Post-Secondary Cert  7. Post-Secondary Diploma  8.Post Graduate 9. Other  |  |
| DB4 | What is your religion? | 1. Islam 2.African Traditionalist3. No religion  4. Christianity 5. Other (Specify)  |  |
| DB5 | Marital status | 1.Single  2.Cohabiting/informal/Consensual  3. Married  4. Divorced  5. Separated 6.Widowed  |  |
| DB6 | Which type of fishing activity do you engagein? | 1. Fish Catching 2. Post-harvesting 3. Maintenance and Repair  4.Porters and Errands  5. Other (Specify)  |  |
| DB7 | How long have you being engaged in this activity in this community? | ………………………………………………. |  |
| DB8 | Nationality | 1.Ghanaian   2. Nigerian   3. Other ECOWAS states (specify) ……………  4.Africa, other than ECOWAS States (specify) ……..  5. European (specify) ………  6.Asian ……………  7. Americas (North, South Caribbean) (specify) ………..  8. Oceania (specify)  …………………………… | **»**→ DB9  **»**→ MS1  **»**→ MS1  **»**→ MS1  **»**→ MS1  **»**→ MS1  **»**→ MS1  **»**→ MS1 |
| DB9 | Which ethnic group do you belong to? | 1.Fante 2.Other Akan  3.Ga Adangbe  4.Mole Dagbani 5.Ewe  6.Guan  7.Other (specify) ……  99. Don’t know  |  |

**SECTION 2:	MOBILITY AND SETTLEMENT PATTERNS AND HIV RISKS**

Now I would like to ask you some questions about mobility and settlement patterns. This will involve questions on the reasons that account for your mobility to other fishing community, and the conditions your settlement in the fishing destination points you visited in the last 12 months

| MS1 | Where is your current place of residence? | ……………………………………… |  |
| --- | --- | --- | --- |
| MS2 | How long have you lived/been in this fishing community? | ……………………………………… |  |
| MS3 | Do you travel to other fishing destinations to engage in fishing related activities | 1.Yes  2. No  | IF ‘NO’ **»**→ AK1 |
| MS4 | In the last 12 months have travelled to other fishing destination to engage in any fishing related activity? | 1.Yes  2. No  | IF ‘NO’ **»**→ MS7 |
| MS5 | If yes, what was the main reason for travelling to other fishing destinations | 1.To buy or sell fish 2.To serve as fish potter 3. To assist Family member’s fishing activity 4. To track fish in other fishing communities  5. To do repair or maintenance work on boats 6. Other {Specify}.. |  |
| MS6 | How many times have you travelled away from this village in the last 12 months? | 1.Once  2.Twice  3.Three times  4. Four times  5. Five times  6. More than five times |  |
| MS7 | How long do you usually stay when you are in other fishing communities? | 1. Day 2. Week 3. Month 4. More than a month 5. Other {Specify}…  |  |
| MS8 | What are the reasons for staying (keeping you) in this/these communities? | ……………………………………… |  |
| MS9 | Does staying away from home affect you in any way? | 1.Yes  2. No  | IF ‘NO’ **»**→ MS11 |
| MS10 | How does staying away from home affect you? | 1. Influences non-regular sexual behaviours  2. Leads to loneliness and isolation  3. Leads to the indulgence in alcohol abuse  4. Other {Specify}… |  |
| MS11 | Where do you normally reside in the other destinations they travel to? | 1. Makeshift wooden structures 2.In friends/relatives home 3. Rented rooms around the fish village 4.Guest house 5.On the boats/canoe |  |
| MS12 | Do you face any other challenges settling in these other fishing communities? | 1.Yes  2. No  | IF ‘NO’ **»**→ AK1 |
| MS13 | If yes, what are these challenges? | ……………………………………….. |  |

**SECTION 4: HIV AND AIDS RELATED KNOWLEDGE AND ATTITUDES OF FISHERS**

I would like to you some few questions about your awareness and knowledge on HIV/STI, and remember there is no right or wrong answer.

| AK1 | Are you aware of Sexually Transmitted Infections (STIs)? | 1.Yes  2. No  |  |
| --- | --- | --- | --- |
| AK2 | Have you ever heard of HIV and/or AIDS? | 1.Yes  2. No  | IF ‘NO’ **»**→ RF1 |
| **Knowledge of HIV Prevention Methods** | | | |
| AK3 | Is there a particular way of protecting oneself from HIV? | 1.Yes  2. No  | IF ‘NO’ **»**→ RF1 |
| AK4 | Indicate the protective method of HIV | A. Using condoms 1.Yes  2. No   B. Abstinence/ Being faithful 1.Yes  2. No   C. Limiting sex to one uninfected partner1.Yes  2. No   D. Avoiding sharing of sharp/piercing objects1.Yes  2. No  | Multiple Response |
| **Sources of Knowledge on HIV and AIDS** | | |  |
| AK5 | What is your source of information on HIV and/or AIDS? | A.Television1.Yes  2. No   B. Radio1.Yes  2. No   C. Newspaper1.Yes  2. No   D. Pamphlet/posters1.Yes  2. No   E. Health Care workers1.Yes  2. No   F. Religious leader1.Yes  2. No   G. Traditional leader1.Yes  2. No   H. Campaign1.Yes  2. No   I. Family member1.Yes  2. No   J. Friend1.Yes  2. No   K. Workplace 1.Yes  2. No   L. Sexual partner 1.Yes  2. No  | Multiple Response |
| AK6 | What is your main source of information on HIV and AIDS? | 1.Television 2.Radio 3. Newspaper 4. Pamphlet/ posters 5. Health Care workers  6. Religious/Traditional leader  7. Community Durbar/Townhall gathering   8. Family member  9. Friend10. Workplace . |  |
| **Comprehensive Knowledge of HIV and AIDS** | | | |
| AK7 | Can a healthy looking person have HIV? | 1.Yes  2. No  |  |
| AK8 | Can you become infected with HIV and AIDS having sexual intercourse without a condom? | 1.Yes  2. No  |  |
| AK9 | Can you become infected with HIV and AIDS from mosquito bites? | 1.Yes  2. No  |  |
| AK10 | Can you become infected with HIV and AIDS through witchcraft? | 1.Yes  2. No  |  |
| AK11 | Can the risk of HIV infection be reduced by having sexual intercourse with only one faithful uninfected partner? | 1.Yes  2. No  |  |
| AK12 | Is AIDS a dangerous disease? | 1.Yes  2. No  |  |
| AK13 | Explain your answer in ??? | ……………………………………… |  |
| AK14 | Are you afraid of AIDS? | 1.Yes  2. No  |  |
| AK15 | Explain your answer | …………………………. |  |
| AK16 | Have you ever had an HIV test? | 1.Yes  2. No  |  |
| AK17 | Would you like to have an HIV test? | 1.Yes  2. No  | IF ‘YES’ **»**→ AK19 |
| AK18 | If no, why would you not want to be tested? | ……………………………………… |  |
| AK19 | If yes, do you know where to have an HIV test? | 1.Yes  2. No  |  |

**SECTION 5: RISKY SEXUAL BEHAVIOURS THAT EXPOSE FISHERS TO HIV**

Now I want us to talk about your sexual activity in the last 12 months; whether you have had sex, whether you have had any sexual encounter (s) with any non-regular partner and the number of such partners. I would also ask you questions about condoms use in your sexual encounters within the period.

| RF1 | Have you had sex in the last 12 months? | 1.Yes  2. No |  |
| --- | --- | --- | --- |
| RF2 | Have you had sexual intercourse with any non-regular partner in the last 12 months? | 1.Yes  2. No  | IF ‘NO’ **»**→ RF4 |
| RF3 | If yes, about how many non-regular partners have you had intercourse with in the last 12 months | 1. One partner 2. Two partners 3. Three partners or more |  |
| RF4 | Have you been using condoms in the last 12 months during sexual intercourse? | 1.Yes  2. No  | IF ‘NO’ **»**→ RF7 |
| RF5 | Was condom used the last time you had sex with a non-regular partner? | 1.Yes  2. No  |  |
| RF6 | If yes, how often? | 1. Always 2. Not always 3. Occasionally 4. Never |  |
| RF7 | IF no what was the main reason for not using a condom the last time you had sex with a non-regular partner? | 1. Partner objected to the use of condoms 2. Condom was not available 3. Trust partner/Didn’t think it was necessary 4. Attractiveness of partner |  |
| RF8 | Have you heard of Fish-for-sex (FFS) transactional relationship? | 1.Yes  2. No  | IF ‘NO’ **»**→ IP1 |
| RF9 | Have you ever engaged in Fish for sex? | 1.Yes  2. No  |  |
| RF10 | In the last 12 months have you engaged in FFS? | 1.Yes  2. No  |  |
| RF11 | Do you use a condom when you have FFS sex? | 1.Yes  2. No  |  |
| RF12 | If yes, what is the main reason for using a condom in FFS sex? | 1.Condom reduces pleasure of sex 2. My partner does not like to use condoms 3. Trust for partner/Didn’t think it was necessary 4. Condom not available |  |
| RF13 | Did you use a condom the last time you had a FFS sex? | 1.Yes  2. No  |  |
| RF14 | If yes what was the main reason for not using a condom the last time you had FFS sex? | ………………………………………………………………. |  |
| RF15 | Why do you engage in FFS transactional relationship? | ………………………………………………………………. |  |
| RF16 | Have you ever encountered stories or reports about single parenthood resulting from FFS relationships in Elmina? | 1.Yes  2. No  |  |
| RF 17 | In your opinion how will you perceive the frequency of occurrence of single FFS parenthood in Elmina? | 1. Never 2. Rarely 3. Sometimes 4. Often 5. Always |  |
| RF 18 | What factors do you believe contribute to the prevalence of FFS relationships in Elmina? | ……………………………………………………………………………… |  |
| RF 19 | What are the perceived effects of single-female parenting? | ……………………………………………………………………………… |  |

**SECTION 6: STRATEGIES FOR CARRYING OUT HIV EDUCATION IN STUDY AREA**

Here I will ask you questions regarding your participation in HIV and AIDS education programmes, reasons for non-participation, the kinds of HIV and AIDS educational interventions currently on-going in the Elmina fishing community and other fishing communities you visited in the last 12 months. This will be very short.

| IP1 | Are you aware of any HIV education in this community? | 1.Yes  2. No  | IF ‘NO’ **»**→ IP3 |
| --- | --- | --- | --- |
| IP2 | If yes, which agency/organisation is implementing this HIV education programme? | …………….. |  |
| IP3 | Have you participated in any HIV programmes in the last 12 months**?** | 1.Yes  2. No  |  |
| IP4 | If you no, why not |  |  |
| IP5 | Have there been any changes in your sexual behaviour due to your participation in any HIV programmes? | 1.Yes  2. No  99. Don’t Know | IF ‘NO’ **END** |
| IP6 | If yes, what changes have occurred in your sexual behaviour due to being part of the HIV program? |  |  |

**Thank you!**

## INTERVIEW GUIDE FOR MALE & FEMALE FOCUS GROUP DISCUSSION (FGD)

A. **BACKGROUND INFORMATION**
1. Physical aspects
   i. How are the areas in the neighborhood different from others?
   ii. What are the patterns of settlement?
   iii. Do both fisher migrants and non-migrants, male and female fishers live in same settlement?
   iv. How do you access water in Elmina and other fishing communities you travel to?
   v. What kind of toilet facilities do you use in Elmina and in the other places you travel to?
   vi. What are your challenges with water, toilet and other sanitation facilities in the fishing communities you know of?
   vii. Generally what are the states of infrastructure and amenities in the fishing communities that you have visited? What are the key challenges that fishers face in fishing communities?
2. Demographic profile of the population
   i. Who makes up the community?  Age, gender, ethnicity, marital status etc.
3. Social structure
   i. Community leaders - formal and informal, Community culture, formal and informal, existing groups, existing institutions. Attitudes and values.
4. Economics (*probe to find out ways in which people earn their living in this community*)
   i. What is the main economic activity? What alternative economic activities exist? What do men and women do for a living? What are the gender roles assigned to both male and female in the fishing activity? Are there inequalities? Please explain
   ii. What is the main economic activity? What alternative economic activities exist? What do men and women do for a living? What are the gender roles assigned to both male and female in the fishing activity? Are there inequalities? Please explain
B. **MOBILITY**
i. How do female/male fishers organize their mobility to the fishing destinations?
ii. Do they travel alone or with other members of their families or close relatives?
iii. What are the situations that keep female/male fishers in other fishing destinations?
iv. What are the settlement patterns (meals, sleeping arrangements and sanitation issues etc.)
v. What risky lifestyles do female/male fishers engage in the fishing community?
vi. Have you heard of fish-for-sex sexual exchanges?
vii. Do fishers in Elmina engage in FFS transactions? If yes, what are men/women’s subjective position regarding ‘fish for sex’ exchange?
viii. How do members of the community see women and men engaged in FFS?
ix. Why do male/female fishers engage in FFS?
x. Do you think FFS can cause HIV among fishers? If yes, why do you say so?
xi. Have you ever encountered stories or reports about single parenthood resulting from FFS relationships in Elmina?
xii. In your opinion how will you perceive the frequency of occurrence of single FFS parenthood in Elmina?
xiii. What factors do you believe contribute to the prevalence of FFS relationships in Elmina?
xiv. What are the perceived effects of single-female parenting?
xv. Generally, what are the main challenges of male/female fishers in this community?
C. **Knowledge, Attitudes and Practices Regarding HIV**
i. Is there anyone here who has not heard of HIV?
ii. What do you know about HIV and AIDS?
iii. What do you think causes AIDS?
iv. How do you know if someone has the virus that causes AIDS?
v. How do people get infected?
vi. Who is at risk of getting infected with HIV, the virus that causes AIDS? Who is not at risk?
vii. In your opinion, what are some of the risky lifestyles in Elmina and the other fishing communities you have visited that might expose fishers to HIV?
viii. How important do you think HIV is compared to other diseases people have?
ix. What can the people in this community do to keep from getting infected?
x. How easy or difficult is it to do these things?
xi. How would people in this area feel if someone in their family or community were infected with HIV?
xii. What or who do you think could be good sources of information about AIDS that people would really listen to? Why?
xiii. What can fisherfolks do to help prevent HIV and AIDS in this community?
xiv. What can fisherfolks do to help people and the families of people who are already HIV-infected or have AIDS?
xv. What sorts of information/training/materials/other support do you need to prevent people from getting infected with HIV and to support people with AIDS and their families?

Are there any other areas you would like to comment on that we haven’t covered today that you think should be included? If not, then I would say thank you for taking part in the focus group discussion.

## INTERVIEW GUIDE FOR GOVERNMENT INSTITUTIONS (GAC, MHA, FD, DEVELOPMENT AND THE MUNICIPAL ASSEMBLY, DEPARTMENT OF GENDER, AND DEPARTMENT OF COMMUNITY DEVELOPMENT

**GHANA AIDS COMMISSION (GAC)**

a. **Introductory Questions**

I would like to start off with some general questions about your involvement with issues on fishers.

i. Is the Ghana Decentralized? Do you have offices in the Districts?
ii. To what extent is GAC involved in community outreach and public education?
iii. What are your methods for engaging with the public on HIV and other STIs?
iv. How effective are your methods and strategies for carrying out HIV education?
v. What are the major steps taken by the Ghana AIDS Commission to improve HIV prevention in the fishing sector?
vi. Does the GAC have any programme that seeks to enhance HIV prevention among fishers?
vii. Does the GAC have any programme that seeks to enhance HIV prevention among fishers the community? Can you provide details on this project or programme?
a. When did you first start with this project? What is the duration of the project?
b. How is it related to fisher mobility and HIV exposure?
c. Are you working in only Elmina fishing community or other communities?
d. If you are working in other communities, please briefly share some of your experiences in respect to knowledge, attitudes and practices of fishers regarding HIV.
e. What are your methods for carrying our HIV information to fishers and the fisher community, if any?
f. What are the challenges you face regarding HIV education in the community?
g. How is the project financed
   b. **Achievement and challenges**
i. What has been the biggest achievement of your engagement with fishers concerning HIV education to date? Why did you select that achievement?
ii. What has been the biggest challenge your engagement with fishers concerning HIV education to date?
    a. Why did you select that challenge?
    b. How have you coped with the challenges?
iii. What do you propose to be done to improve knowledge, attitudes and practices of fishers regarding HIV and other risky behaviours that increase fishers’ exposure to HIV?

Now I would like to talk about human rights, legislations and regulations on HIV.

i. Does the country have laws and regulations that protect people living with HIV against discrimination? (*Such as general non-discrimination provisions or provisions that specifically mention HIV, focus on schooling, housing, employment, health care etc*.)
ii. Does the country have non-discrimination laws or regulations which specify protections for vulnerable sub-populations such as fishers?
iii. Has the Government, through political and financial support, involved most at-risk populations in governmental HIV-policy design and programme implementation?
iv. At present the HIV prevalence rates in fishing communities in Ghana is known. Does the GAC or government have any future plans to improve data on HIV among high prevalent HIV sub-populations such as fishers? Briefly give an account of any such intentions
v. Are there programmes to change societal attitudes of stigmatization associated with HIV and AIDS to understanding and acceptance? If Yes, what types of programmes.

**FISHERIES DEPARTMENT (FD)**

a. **Introductory Questions**

I would like to start off with some general questions about your involvement with issues on HIV among fishers.

i. Is HIV having any effects on the fisheries sector? What are the effects and how has it impacted on fisheries management and development?
ii. What are the major steps taken by the Fisheries Department to improve Fisheries management and development?
iii. Does the Department have any programme that seeks to enhance HIV prevention among fishers? Can you provide details on this project or programme?
     a. When did you first start with this project? What is the duration of the project?
     b. How is it related to fisher mobility and HIV exposure?
     c. Are you working in only Elmina fishing community or other communities?
     d. If you are working in other communities, please briefly share some of your experiences in respect to knowledge, attitudes and practices of fishers regarding HIV.
     e. What are your methods for carrying our HIV information to fishers and the fisher community, if any?
     f. What are the challenges you face regarding HIV education in the community?
     g. How is the project financed
     b. **Achievement and challenges**
i. What has been the biggest achievement of your engagement with fishers concerning HIV education to date? Why did you select that achievement?
ii. What has been the biggest challenge your engagement with fishers concerning HIV education to date?
    a. Why did you select that challenge?
    b. How have you coped with the challenges?
iii. What do you propose to be done to improve knowledge, attitudes and practices of fishers regarding HIV and other risky behaviours that increase fishers’ exposure to HIV?

**MUNICIPAL HEALTH ADMINISTRATION (MHA)**

a. **Introductory Questions**

I would like to start off with some general questions about your involvement with issues on fishers.

b. **Awareness, knowledge, attitudes and practices of fishers regarding HIV**

i. Is HIV risk a major concern among fishers in Elmina?
   a. Do the fisherfolks in Elmina consider HIV as a major risk and threat to their development? Please share your thoughts on this.
   b. How would you describe the level of awareness and knowledge of fishers regarding HIV?
   c. What are your observations on the attitudes and practices of fishers regarding HIV in this community and the fishing communities you operate in?
ii. What are the major concerns regarding HIV risk behaviours?
    a. Is HIV risk denial an issue here?
    b. Are fishers engaged in excessive alcohol drinking?
    c. Do fishers engage in drugs?
    d. What are the issues concerning fishers and sex networking (casual sex, multiple sexual relationships, Sex with sex workers and fish-for-sex transactions) – (*Take the issues one after the other and probe for details*).
iii. Is the Health Directorate currently working on any project concerning the protection of fishers against HIV and other STIs? Is the project offered in this community/and or other communities? Can you provide details on this project or programme?
     a. When did you first start with this project? What is the duration of the project?
     b. How is it related to fisher mobility and HIV exposure?
     c. Are you working in only Elmina fishing community or in other fishing communities?
     d. If you are working in other communities, please briefly share some of your experiences in respect to knowledge, attitudes and practices of fishers regarding HIV.
     e. What are your methods for carrying our HIV information to fishers and the fisher community, if any?
     f. Do fishers from the community visit your service? How accessible are your services (very easy, easy, very difficult, difficult, impossible). What do fishers use your facility for?
     g. What are the challenges you face regarding HIV education in the community?
     h. How is the project financed
iv. Do you have incentives in place to ensure safe sex among fishers?
    a. Do you distribute free condoms, or provide free counselling on HIV and STIs.
    b. Do you, or any other local service, provide voluntary counselling and testing services (VCT) in the community? If yes what is the name of the organisation?
    c. **Achievement and challenges**
i. What are the key strengths of your organisation and the service it provides?
ii. What difficulties does the institution experience in delivering services or what stops it from meeting the needs of the community? (*Probe: lack of resources, inaccessibility, loss of staff, poverty etc*.)
iii. What has been the biggest achievement of your engagement with fishers concerning HIV education to date? Why did you select that achievement?
iv. What has been the biggest challenge your engagement with fishers concerning HIV education to date?
    a. Why did you select that challenge?
    b. How have you coped with the challenges?
    c. Is there anything else you would like to tell me about the HIV and AIDS related services you provide in relation to fishing communities, or about the groups of people from fishing communities who use your service and how they are being affected by HIV/AIDS?
v. What do you propose to be done to improve knowledge, attitudes and practices of fishers regarding HIV and other risky behaviours that increase fishers’ exposure to HIV?

**KOMENDA EDINA EGUAFO ABIREM (KEEA) MUNICIPAL ASSEMBLY**

a. **Introductory Questions**

I would like to start off with some general questions about your involvement with issues on HIV among fishers.

i. Is HIV a major concern in the Elmina fishing community? Please share your thoughts on it.
ii. Do you feel HIV is having any effects on the fisheries sector? What are the effects and how has it impacted on fisheries management and development?
iii. Does the Assembly have any programme that seeks to enhance HIV prevention among fishers? Can you provide details on this project or programme?
a. When did you first start with this project? What is the duration of the project?
b. How is it related to fisher mobility and HIV exposure?
c. What are your methods and strategies used by the Assembly for carrying our HIV information to fishers and the fisher community, if any?
d. What are the challenges you face regarding HIV education in the community?
e. Does the Assembly make budgetary provision for HIV programmes in the Assemblies budget? What are your sources of funding for HIV interventions? What percentage of the budget is allocated for HIV programmes? What are the specific activities captured in the budget of the Assembly?
   b. **Achievement and challenges**
i. What has been the biggest achievement of the Assembly’s engagement with fishers concerning HIV education to date? Why did you select that achievement?
ii. What has been the biggest challenge your engagement with fishers concerning HIV education to date?
    a. Why did you select that challenge?
    b. How have you coped with the challenges?
iii. What do you propose to be done to improve knowledge, attitudes and practices of fishers regarding HIV and other risky behaviours that increase fishers’ exposure to HIV in the KEEA Municipality?

**DEPARTMENT OF COMMUNITY DEVELOPMENT AND DEPARTMENT OF GENDER**

a. **Introductory Questions**

I would like to start off with some general questions about your involvement with issues on fishers.

b. **Awareness, knowledge, attitudes and practices of fishers regarding HIV**

i. Is HIV risk a major concern among fishers in Elmina?
   a. Do the fisherfolks in Elmina consider HIV as a major risk and threat to their development? Please share your thoughts on this.
   b. How would you describe the level of awareness and knowledge of fishers regarding HIV?
   c. What are your observations on the attitudes and practices of fishers regarding HIV in this community and the fishing communities you operate in?
ii. Is the Department of Community Development/Department of Gender currently working on any project concerning the protection of fishers against HIV and other STIs? Is the project offered in this community/and or other communities? Can you provide details on this project or programme?
    a. When did you first start with this project? What is the duration of the project?
    b. How is it related to fisher mobility and HIV exposure?
    c. Are you working in only Elmina fishing community or in other fishing communities?
    d. If you are working in other communities, please briefly share some of your experiences in respect to knowledge, attitudes and practices of fishers regarding HIV.
    e. What are your methods for carrying our HIV information to fishers and the fisher communities, if any?
    f. What are the challenges you face regarding HIV education in the community?
    g. How is the project financed

iii. What is the general attitude toward parenting in Elmina, particularly regarding single parenthood and FFS relationships?

c. Have you ever encountered stories or reports about single parenthood resulting from FFS relationships in Elmina?
d. In your opinion how will you perceive the frequency of occurrence of single FFS parenthood in Elmina?
e. What factors do you believe contribute to the prevalence of FFS relationships in Elmina?
f. What are the perceived effects of single-female parenting?
g. **Achievement and challenges**

i. What are the key strengths of your organisation and the service it provides?
ii. What difficulties does the institution experience in delivering services or what stops it from meeting the needs of the community? (*Probe: lack of resources, inaccessibility, loss of staff, poverty etc*.)
iii. What has been the biggest achievement of your engagement with fishers concerning HIV education to date? Why did you select that achievement?
iv. What has been the biggest challenge your engagement with fishers concerning HIV education to date?
v. What do you propose to be done to improve knowledge, attitudes and practices of fishers regarding HIV and other risky behaviours that increase fishers’ exposure to HIV?

## INTERVIEW GUIDE FOR NON-GOVERNMENTAL ORGANISATIONS (FOUNDATION FOR BUILDERS AND KIDS CLUBS & HUMAN SERVICE TRUST FOUNDATION)

a. **Introductory Questions**

i. Are you working on any project concerning movements of fishers and their protection against HIV and other STIs? Can you provide details on this project or programme?
   a. When did you first start with this project?
   b. How is it related to fisher mobility and HIV exposure?
   c. Are you operating in only Elmina fishing community or in other fishing communities?
   d. If you are working in other fishing communities, please briefly share some of your experiences in respect to knowledge, attitudes and practices of fishers regarding HIV.

**b. Awareness, knowledge, attitudes and practices of fishers regarding HIV**

ii. Is HIV risk a major concern among fishers in Elmina?
    a. Do the fisherfolks in Elmina consider HIV as a major risk and threat to their development? Please share your thoughts on this.
    b. How would you describe the level of awareness and knowledge of fishers regarding HIV?
    c. What are your observations on the attitudes and practices of fishers regarding HIV in this community?
iii. What are the major concerns regarding HIV risk behaviours?
     a. Is HIV risk denial an issue here?
     b. Are fishers engaged in excessive alcohol drinking?
     c. Do fishers engage in drugs?
     d. What are the issues concerning fishers and sex networking (casual sex, multiple sexual relationships, Sex with sex workers and fish-for-sex transactions) – (*Take the issues one after the other and probe for details*).

iv.	What is the general attitude toward parenting in Elmina, particularly regarding single parenthood and FFS relationships?

a.	Have you ever encountered stories or reports about single parenthood resulting from FFS relationships in Elmina?

b.	In your opinion how will you perceive the frequency of occurrence of single FFS parenthood in Elmina?

c.	What factors do you believe contribute to the prevalence of FFS relationships in Elmina?

d.	What are the perceived effects of single-female parenting?

v. What are your methods for carrying our HIV information to fishers and the fisher community, if any?

e. What are the challenges you face regarding HIV education in the community?
f. How have you coped with the challenges?
g. What have been your success stories in your work with the fishers?
h. What will you recommend to enhance your work?

vi. Do you have incentives in place to ensure safe sex among fishers?
    a. Do you distribute free condoms, or provide free counselling on HIV and STIs.
    b. What other incentives do you have?
    c. **Achievement and challenges**
i. What has been the biggest achievement of your engagement with fishers concerning HIV education to date? Why did you select that achievement?
ii. What has been the biggest challenge your engagement with fishers concerning HIV education to date? Why did you select that challenge?
iii. What do you propose to be done to improve knowledge, attitudes and practices of fishers regarding HIV and other risky behaviours that increase fishers’ exposure to HIV?

## INTERVIEW GUIDE FOR KEY INFORMANT INTERVIEWS COMMUNITY LEADERS (OPINION LEADERS)

1. **Fisher mobility and settlement**

i. How long have you lived in this community? Please kindly tell me about the fishing activity in this area?
ii. Why do fishers move to fish in this area, and where from?
    a. Why do they leave and where do they go to?
    b. Since when have they been migrating to this community?
    c. How is their migration organised? Do they migrate alone, with families or in groups?
    d. What position do they have in the receiving settlement?
    e. How do they get permission to stay and access to the fishing grounds?

Now I will like us to talk about fishers in this community and their knowledge, attitudes and practices regarding HIV.

2. **Awareness, knowledge, attitudes and practices of fishers regarding HIV**

iii. Is HIV risk a major concern among fishers in Elmina?
     a. Do the fisherfolks in Elmina consider HIV as a major risk and threat to their development? Please share your thoughts on this.
     b. How would you describe the level of awareness and knowledge of fishers regarding HIV?
     c. What are your observations on the attitudes and practices of fishers regarding HIV in this community?
     d. How do fisherfolks and members of this community get their information on HIV? Are these sources effective in providing adequate information on the disease?
     e. What have been your overall impressions of HIV education in the community?
     f. Has your own knowledge, attitudes or practices on HIV changed since you heard of HIV? If yes:
        i. What has changed in terms of your knowledge, attitudes or practices?
        ii. What do you think caused these changes to happen?
        iii. Can you say same of other members of the community?
iv. What are the major concerns regarding HIV risk behaviours?
    a. Is HIV risk denial an issue here? Do you think fishers ignore the danger posed by HIV due to the daily dangers they face at sea?
    b. Are fishers engaged in excessive alcohol drinking? Do fishers engage in drugs?
    c. What are the issues concerning fishers and sex networking (casual sex, multiple sexual relationships, Sex with sex workers and fish-for-sex transactions) – (*Take the issues one after the other and probe for details*)
    d. What are people’s subjective position regarding ‘fish for sex’ exchange? What do community members feel about FFS practice? How do members of the community see women and men engaged in FFS? Is it likely women who engage in FFS could face social exclusion or stigmatization?
    e. Have you observed any changes in sexual behaviours since the outbreak of HIV?
    f. Is HIV one of the factors that have contributed to the changes?
    g. What changes in sexual behaviours have you observed in the community since the outbreak of HIV? What actions caused these changes?

v.	What is the general attitude toward parenting in Elmina, particularly regarding single parenthood and FFS relationships?

a.	Have you ever encountered stories or reports about single parenthood resulting from FFS relationships in Elmina?

b.	In your opinion how will you perceive the frequency of occurrence of single FFS parenthood in Elmina?

c.	What factors do you believe contribute to the prevalence of FFS relationships in Elmina?

d.	What are the perceived effects of single-female parenting?
